# The Entropy-Based Time Domain Feature Extraction for Online Concept Drift Detection

**DOI:** 10.3390/e21121187

**Published:** 2019-12-02

**Authors:** Fengqian Ding, Chao Luo

**Affiliations:** 1 School of Information Science and Engineering, Shandong Normal University, Jinan 250014, China; wygtsmile1020@gmail.com; 2 Shandong Provincial Key Laboratory for Novel Distributed Computer Software Technology, Jinan 250014, China

**Keywords:** concept drift, time series, entropy, statistical process control

## Abstract

Most of time series deriving from complex systems in real life is non-stationary, where the data distribution would be influenced by various internal/external factors such that the contexts are persistently changing. Therefore, the concept drift detection of time series has practical significance. In this paper, a novel method called online entropy-based time domain feature extraction (ETFE) for concept drift detection is proposed. Firstly, the empirical mode decomposition based on extrema symmetric extension is used to decompose time series, where features in various time scales can be adaptively extracted. Meanwhile, the end point effect caused by traditional empirical mode decomposition can be avoided. Secondly, by using the entropy calculation, the time-domain features are coarse-grained to quantify the structure and complexity of the time series, among which six kinds of entropy are used for discussion. Finally, a statistical process control method based on generalized likelihood ratio is used to monitor the change of the entropy, which can effectively track the mean and amplitude of the time series. Therefore, the early alarm of concept drift can be given. Synthetic data sets and neonatal electroencephalogram (EEG) recordings with seizures annotations data sets are used to validate the effectiveness and accuracy of the proposed method.

## 1. Introduction

The study of time series has strong theoretical significance and application value in real life. Due to its practical importance, the works related to the applications of time series are widely used in finance, engineering, medicine, and other fields [1,2,3,4]. The time series deriving from real life are normally non-stationary, which means the contents of sequence data would change over time due to various factors. For example, EEG data of a patient with epilepsy would be considerably different in normal state and during the attack, which leads to distinct contents of bio-information time series. These changes are known as concept drift, which widely exists in various kinds of time series data [5,6]. The study of concept drift of time series has the practical implications. For instance, the prediction of time series is always a hot topic in this community and various studies have been proposed [7,8,9]. However, the existing prediction models commonly depended on the specific data, which means a new volatility pattern of time series could greatly affect the prediction performance. The root cause of the above case is the existence of concept drifts. Since the prediction models are trained based on the original concepts of time series, with the emergence of concept drift, it cannot be suitable for the current situation such that the prediction accuracy would be affected. Therefore, how to effectively detect the concept changes of time series is of significance. 

Generally, concept drift detection methods can be divided into two types [5], one is explicit detection methods, i.e., supervised detection methods, and the other is implicit detection methods, i.e., unsupervised detection methods. From the perspective of probability, the explicit detection methods regard the concept drift as the change of the joint probability distribution P(X,Y) of the sample data X and its corresponding label Y, and the implicit detection methods are to track the change of the sample data distribution P(X) [5]. From another point of view, explicit detection methods usually need base-learners to deal with classification problems, and directly determine the occurrence of drifts by monitoring whether the performance indicators of base learner classification (such as classification error rate) reach a threshold [10,11,12]. When dealing with concept drift, these methods usually discard the previous base-learners and replace them with a new base-learner. For some ensemble learning methods [13,14], they will be according to the performance of each base-learner to decide whether to add a new base-learner, reduce an existing one, or adjust their corresponding weights. Implicit detection methods do not need data labeling. By extracting and transforming the features of data, they monitor the changes of data features to achieve the purpose of concept drift detection [15,16], where the so-called changes generally include statistical characteristics of data, data distribution, or some particular metrics.

Even though many approaches related to the detection of concept drifts of time series have been proposed in recent years [17,18,19], some problems are still open. On the one hand, most of the existing detection algorithms are based on the performance indicators of the classifiers. However, time series data are difficult to be marked in the real environment such that the absence of ground truth is an unavoidable problem. On the other hand, some concept drift detection methods are based on the assumption of independent data. Therefore, due to the particularity of time series data, it is impractical to apply the existing models without any modification. In addition, in the real environment, considering the influence of noises in the time series, the obtained data is also difficult to be learnt directly [20]. 

In order to solve the difficulties mentioned above, in this paper, a novel unsupervised algorithm is proposed for the online time series concept drift detection. Firstly, an empirical mode decomposition (EMD) method [21] based on extrema symmetric extension is used to decompose time series. After decomposition, a series of intrinsic mode functions (IMFs) containing different time scales of the original signals can be obtained, where various features of time series in different scales can be revealed. Furthermore, entropy methods have been used to measure the structure and complexity of time series, where the structural characteristics of IMFs with different frequencies can be analyzed. Compared with directly monitoring from the original signals, the obtained data has higher signal-to-noise ratio and intuitiveness. When concept drifts occur, the changes of time series would result in the fluctuations of entropy values. In order to detect the changes, a generalized likelihood ratio (GLR) based statistical process control algorithm [22] is used. This method calculates the statistical characteristics of the data in each sliding window and compares with a given threshold to judge the breakpoint, so as to determine the location of the concept drift. The main contributions are summarized as follows:

- A novel unsupervised algorithm is proposed for online time series concept drift detection, which can effectively detect the occurrence of concept drift in streaming data by capturing the fine structures of data in different time scale.
- Entropy methods are used to capture the changes of intrinsic structures of the original sequence in different time domains, where multiple application scenarios are discussed according to the characteristics of entropies in detail.
- A statistical process control method based on GLR is designed to monitor the changes of the obtained entropy information, which can determine the concept drift in time and reduce the false alarms.

The rest of the paper is organized as follows: The second part presents the literature review; the third part is the introduction of the proposed algorithm entropy-based time domain feature extraction (ETFE), where the principle and implementation are included; the fourth part is the related experiments, which include the performance evaluation of the proposed method in synthetic data and real data; the fifth part is the conclusion and prospect of our work.

## 2. Related Works

In recent years, some theoretical results have been proposed to tackle with the concept drifts in time series. In order to solve the problem that real time series data are difficult to be labeled due to the characteristics of flow patterns and high frequencies, Cavalcante [23] proposed an explicit drift method by exploring the influence of concept drift in financial time series on prediction accuracy, where ELM [24] was used as prediction method, DDM [10] and ECDD [11] were used as drift detectors. In the following work, Cavalcante proposed a new concept drift detection method called feature extraction drift detection (FEDD) [17], which determined the presence of concept drifts by detecting the temporal characteristics of the time series, and it can also provide a better explanation of temporal evolution than monitoring prediction accuracy. 

In order to deal with the influence of time dependence of time series on concept drift detection, Guajardo [25] proposed a support vector machine regression model based on seasonal pattern to predict time series. The idea of this method was to divide the data in a sliding window into training set and test set. When the sliding window moved forwards, latest data were used to retrain the model. The size of the sliding window was adjusted according to the seasonal pattern of the time series to adapt to the characteristics of the data in the current time period. In this way, the model structure can take the latest data information into account, but for real time series without predefined seasonal patterns, the cycle of acquiring seasonal patterns will not be practical.

Costa et al. [19] dealt with the concept drift of time series by decomposing the time series into deterministic components consisting of non-independent observations and stochastic components consisting of independent observations. In order to eliminate the time dependence in deterministic components, Taken’s immersion theory was used to decompose deterministic components into independently and identically distributed data. In this way, both deterministic and stochastic components were subjected to independent and identical distribution, and the constructed model from these data can be more stable.

In this paper, a novel unsupervised algorithm is proposed for the online time series concept drift detection. Compared with the existing detection methods, the novelty and innovation brought by this approach is that, based on IMFs revealing the original signals, entropy methods are used to capture the changes of intrinsic structures of the original sequence in different time domains, where the extracted features have higher signal-to-noise ratio. Furthermore, the statistical control process can effectively determine the occurrence of concept drift and reduce the false alarms.

## 3. Model

**Figure 1:**
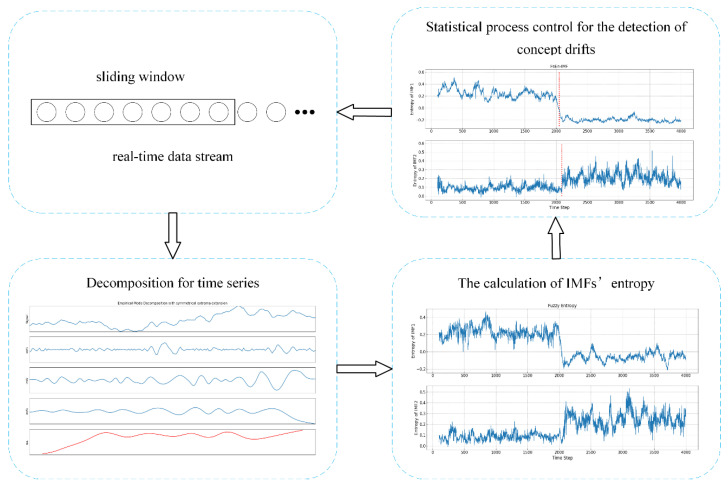
Entropy-based time domain feature extraction (ETFE) model framework.

In this section, the ETFE method is to be introduced in detail, which is an online unsupervised concept drift detection algorithm for time series. Since the existence of noise and abnormal interferences in the original time series, it is difficult to directly detect concept drift from the original data [26]. Based on EMD with the extrema value symmetric extension, IMFs obtained by decomposing the original time series can extract the features of time series in various time scales. Since the high frequency IMF is more sensitive and the low frequency IMF can reveal the overall trend, by combination of different IMFs, the early alarm for concept drift can be achieved. Entropy, as a measure of complexity, can quantify the structure and fluctuation scales of the time series. Therefore, the drifts will be reflected in the changes of entropy, i.e., the changes of entropy information can be detected through statistical control process. Generally, the proposed method mainly consists of three parts: firstly, an EMD based on extrema symmetric extension is used to decompose the original time series; secondly, the features of IMFs in different time scales are calculated by using entropies; thirdly, the IMF-Entropy values are monitored by a GLR-based statistical control process algorithm such that the occurrence of concept drift can be detected. The flow chart of the whole model is shown in Figure 1.

### 3.1. The Decomposition for Time Series

EMD is a method proposed by Huang et al. [21], which can decompose the signals into different IMFs according to the time scales of the data. Each IMF has a clear physical meaning and contains features of the original data. EMD can be used to analyze non-linear and non-stationary signal sequences with high signal-to-noise ratio and time-frequency focusing. In addition, EMD method has strong local representativeness and can be applied to tackle with time-varying signals. The advantages of EMD are the reason it is often used in time series analysis in the fields of medicine, industrial production, and financial derivatives [27,28].

However, the process of EMD is normally affected by the endpoint effect, and the divergent results will gradually pollute the data inward, resulting in distortion of the results [29]. Different methods have been proposed for handling with endpoint effect [30], where the symmetrical extrema extension can be taken as the primary method because of its small impact on the final result [31]. The basic idea of extrema symmetric extension is that, before the cubic spline interpolation of signals is carried out, the relationships between the maximum, the minimum, and the endpoint are judged first, and the extrema symmetric extensions of the data at both ends are implemented, respectively. Based on the previous works, the decomposition of time series can be carried out as follows:

1. Let x(t),t=1,2,…,l present the time series. x(t) contains *M* local maximums and *N* local minimums, and their indexes are denoted as $I_{m}\left(i\right),i=1,2,\ldots ,M$ and $I_{n}\left(i\right),i=1,2,\ldots ,N$, respectively. In this way, the corresponding local maximum and local minimum are $Ui=x\left(I_{m}\left(i\right)\right),i=1,2,\ldots ,M$ and $Vi=x\left(I_{n}\left(i\right)\right),i=1,2,\ldots ,N$.
2. Start from the left side. When $I_{m}\left(1\right)<I_{n}\left(1\right)$, if the value of the left end point is larger than the first local minimum value, that is x(1)>V(1), then the local maximum value point $I_{m}\left(1\right)$ is used as the center of symmetry to extend *d* units to left. The time indexes and values of the extension sequence are:
  $$k=2I_{m}\left(1\right)-i,\;x\left(k\right)=x\left(i\right),\;i=I_{m}\left(1\right)+1,\ldots ,I_{m}\left(1\right)+d$$
3. When $I_{n}\left(1\right)<I_{m}\left(1\right)$, if the value of the left end point is smaller than the first local minimum value, that is x(1)<U(1), then the local minimum value point $I_{n}\left(1\right)$ is used as the center of symmetry to extend *d* units to left. The time indexes and values of the extension sequence are:
  $$k=2I_{n}\left(1\right)-i,x\left(k\right)=x\left(i\right),\;i=I_{n}\left(1\right)+1,\ldots ,I_{n}\left(1\right)+d$$
4. When x1<V(1) or x(1)>U(1), the left endpoint is used as the symmetric center to extend *d* units to the left, and the time indexes and values of the extension sequence are obtained as follows:
  k=2−i, xk=x(i), i=2,…,d
5. Extend the right endpoint in the same way.
6. Find out all local maximum points and local minimum points in the sequence xt after extension, and fit the upper envelope u(t) of the maximum points and the lower envelope v(t) of the minimum points by cubic spline interpolation. Then, the original sequence is between the upper envelope and the lower envelope. Subsequently, by calculating the mean p(t) of the upper envelope and the lower envelope, the original sequence can be converted into a new sequence h(t):
  p(t)=(u(t)+v(t))/2h(t)=x(t)−p(t)
7. Check if the obtained h(t) meets the following conditions:
  (1) The number of local extremum points and the number of zero crossing points is equal or the difference is at most 1.
  (2) The average of the envelopes of the local maximum and the local minimum is zero.
  If the above two conditions are satisfied, the obtained h(t) is called as *s*-th IMF, where *s* indicates the number of repeats of steps 6 and 7. Then, the obtained h(t) is denoted by $h_{s}\left(t\right).$ And if not, replace x(t) with h(t). Repeat step 6 until h(t) meets the above criteria.
8. Residual r(t) is the difference between h(t) and xt obtained in step 7 and then x(t) is replaced by r(t) to calculate the next IMF. The steps 6–7 are repeated *f* times until the obtained *f*-th residual is a monotonic function. In this way, the original time series xt is represented in the following form:
  $$x\left(t\right)=\overset{f}{\underset{i=1}{\sum }}h_{i}\left(t\right)+r_{f}\left(t\right)$$
9. Delete the data of the extension part and retain only the data decomposed from the original part.

### 3.2. The Calculation of IMFs’ Entropy 

Since the noise and disturbance existing in time series, the changes of time-domain characteristics of time series are difficult to be captured by directly extracting information from raw sequence data [32]. When the contents of time series change, in order to quantify the change degrees and track the processes from different time scales, EMD with extrema symmetric extension is first used to decompose time series adaptively so as to get IMFs in different time domains. Then, the entropy of IMFs is calculated such that the time series can be monitored from the angle of time domain characteristics in various time scales. 

Approximate entropy (ApEn) is a kind of statistical measuring for the complexity of time series, which can be applied in the non-linear and non-stationary data with high noise [33]. Generally, the approximate entropy can be calculated as follows:

1. Time series x(1),x(2),…,x(l) are provided, and a threshold r (usually chosen as 0.2 std, where std is the standard deviation of the original sequence) for similarity comparison and a metric γ (usually chosen as 2 or 3) for defining the length of the reconstructed sequence.
2. The original sequence is reconstructed to obtain l−γ+1 subsequences X(1),X(2),…,X(l−γ+1). Among them, subsequence X(i)=x(i),x(i+1),…,x(i+γ−1).
3. The distance $d_{\gamma }\left[X\left(i\right),X\left(j\right)\right]$ between two reconstructed vectors X(i) and X(j) is calculated, where $d_{\gamma }$ is determined by the maximum difference of the corresponding position elements in the two vectors.
4. Count the number of vectors satisfying the following conditions, and calculate the ratio between the number and the total subsequence data length:
  $$C_{i}^{\gamma }\left(r\right)=\frac{num\left[d_{\gamma }\left(X\left(i\right),X\left(j\right)\right)<r\right]}{l-\gamma +1}$$
  This process is called the template matching process of X(i), and $C_{i}^{\gamma }\left(r\right)$ represents the matching probability between any X(j) and template X(i).
5. Calculate the average similarity rate:
  $$\phi_{\gamma }\left(r\right)=\frac{\sum_{i=1}^{L-\gamma +1}log\left(C_{i}^{\gamma }\left(r\right)\right)}{l-\gamma +1}$$
6. According to steps 1–5 above, the average similarity rate is calculated when the length of subsequence is divided by γ + 1.
7. Calculate the approximate entropy:
  $$ApEn(l,\gamma ,r)=\phi_{\gamma }\left(r\right)-\phi_{\gamma +1}\left(r\right)$$

It can be seen from the calculation process of ApEn that, when the difference between two subsequences is large, the number that satisfies $d_{\gamma }\left[X\left(i\right),X\left(j\right)\right]\leq r$ will be small, and the amount of information corresponding to it will be large. Meanwhile, ApEn has some shortcomings. As a result of the existence of self-matching, it shows a bias towards regularity. There is a lack of relative consistency between approximate entropy values calculated by different parameter combinations, and it is also sensitive to the length of data sets. 

Sample entropy (SampEn) [34] is an improvement of ApEn. The calculation process is similar to that of ApEn, but some shortcomings of ApEn have been overcome. SampEn is based on the model of logarithmic function. In order to avoid the occurrence of ln(0), when calculating the distance between reconstructed vectors, the process of self-matching is eliminated such that ApEn exhibits good relative consistency and is independent of the length of the data set.

Different from SampEn, fuzzy entropy (FuzzEn) [35] introduces an exponential function, namely a fuzzy membership function, to measure the similarity between two sequences. The fuzzy membership function is continuous and therefore, it ensures that the FuzzEn value is stable and does not mutate. Meanwhile, it also ensures the maximum self-similarity value of the sequence. In addition, the change of parameters of FuzzEn has little effect on the computed results.

Although SampEn, ApEn, and FuzzEn can be used to measure the complexity of time series, they ignore the time dependence of elements in time series. Permutation Entropy (PeEn) [36] is a measure of time series complexity from the perspective of intrinsic structure of time series. It calculates the PeEn value by comparing the adjacent values and mapping them to ordered patterns to obtain the frequency of each permutation.

In the definition of PeEn, when extracting ordered patterns for each time series, no other information is retained except the ordered structure, such as the magnitude of time series information. This may lead to the same PeEn value for time series with different amplitude scales or fluctuation patterns. Weighted Permutation Entropy (WPeEn) [37] can better capture abrupt changes in time series by assigning different weights to sequences according to fluctuation sizes. It is calculated in a similar way to the PeEn method, but the WPeEn can better detect some mutations and amplitude changes by introducing the variance of the sequence as a weight. 

Increment entropy (IncrEn) is a new measure of time series complexity in recent years [38], the definition of which is similar to PeEn. But, in the calculation of IncrEn, the relationship between two adjacent elements in time series is expressed by two variables, one of which represents the direction of fluctuation and the other represents the magnitude of fluctuation. In this way, a time series is characterized by the direction and amplitude of fluctuations between adjacent elements, and then the frequency of the characteristic vectors is counted to quantify the complexity of the time series. Additionally, IncrEn also introduces a parameter to indicate the precision of the fluctuation amplitude. If the precision is set too large, it will be sensitive to noise, and if the setting is too small, the information expressed will be less. Therefore, the choice of parameter will affect the value of IncrEn to some extent.

Therefore, in order to comprehensively analyze the application of entropy in the concept drift detection, various entropy methods, including the six entropies above, have been conducted and the comparative results have been discussed. 

### 3.3. Statistical Process Control for the Detection of Concept Drifts

From the discussion results of IMF-Entropy, it can be seen that, when concept drift occurs, the calculation results of IMFs’ entropy change in the values of the mean, variance, or both. In order to monitor its changes, a statistical process control (SPC) model based on GLR [39] is used. In the existing works, the traditional concentration inequality such as Hoeffding’s Inequality [40], Bernstein’s Inequality [41], can only capture the deviation between the mean and its expectation, but it is difficult to work in the situation where the mean changes are slight but the fluctuation is obvious. Therefore, the statistical process control model is applied, where changes existing in both mean and variance can be detected.

We simulate a process as follows:

$$x\left(i\right)\sim \{\begin{array}{c} N\left(\mu_{1},\sigma_{1}^{2}\right)\;if\;i\leq \tau \\ N\left(\mu_{2},\sigma_{2}^{2}\right)\;if\;i>\tau \end{array}$$

where x(1),x(2),…,x(i),… are the successive observations. In this process, the mean, the variance, or both, of the processes change after the time point τ.

It is assumed that the change point ϑ, and the current time step is q, where 0<ϑ<q, the GLR test statistic is defined as:

$$GLR=\vartheta log\frac{S_{0,q}}{S_{0,\vartheta }}+\left(q-\vartheta \right)log\frac{S_{0,q}}{S_{\vartheta ,q}}$$

where $S_{i,j}=V_{i,j}/\left(j-i\right)$, and $V_{i,j}$ is the variance of the sequence x(i+1),…,x(j). 

According to [39], in the case of no shift, this statistic has an asymptotic chi-squared distribution with 2 degrees of freedom. The quality of this approximation can be improved substantially by dividing the Bartlett correction factor, so as to make the expectation of the GLR equal to the degrees of freedom:

$$G_{\vartheta ,q}=\left(\vartheta log\frac{S_{0,q}}{S_{0,\vartheta }}+\left(q-\vartheta \right)log\frac{S_{0,q}}{S_{\vartheta ,q}}\right)/C\;C=1+\frac{11}{12}\left(\frac{1}{\vartheta }+\frac{1}{q-\vartheta }-\frac{1}{q}\right)+\left(\frac{1}{\vartheta^{2}}+\frac{1}{{\left(q-\vartheta \right)}^{2}}-\frac{1}{q^{2}}\right)$$

If there is no prior knowledge to determine the location of the change point, the max $G_{\vartheta ,q}$ can be found through the GLR test process at all possible points, yielding $G_{max,q}=max_{\vartheta }G_{\vartheta ,q}$, and then the drift can be determined by comparing with the control threshold. The whole continuous SPC process is as follows:

1. When the number of consecutive observations reaches a predefined number, $G_{max,q}$ is calculated.
2. If $G_{max,q}\leq \delta_{q}$, where $\delta_{q}$ is an appropriate control threshold, it means that there is insufficient evidence for the occurrence of shifts of variance and mean in the data stream.
3. If $G_{max,q}>\delta_{q}$, it means that there is evidence for the occurrence of shifts of variance and mean in the data stream.

In the implementation of GLR algorithm, the space complexity is not high. Only two arrays are needed for the calculation. One array is the sum of the whole data $W_{q}=\sum_{i=1}^{q}x\left(i\right)$, and the other array is the sum of squared deviations from the moving mean $P_{0,q}$. The calculation of two arrays can be quickly updated by the following recursive formulas:

$$W_{q+1}=W_{q}+x\left(q+1\right)\;P_{0,q+1}=P_{0,q}+q{\left(x\left(q+1\right)-W_{q}/q\right)}^{2}/\left(q+1\right)$$

GLR test statistics can be easily calculated:

$${\bar{X}}_{i,\vartheta }=\left(W_{\vartheta }-W_{i}\right)/\left(\vartheta -i\right)\;P_{i,\vartheta }=P_{0,\vartheta }-P_{0,i}-i\left(\vartheta -i\right)/\vartheta {\left({\bar{X}}_{0,i}-{\bar{X}}_{i,\vartheta }\right)}^{2}$$

Although the computational speed of the statistics required for GLR test is fast, the process of finding the appropriate breakpoint ϑ to maximize $G_{\vartheta ,q}$ will become a burden because of the increasing amount of streaming data. So, the Willsky–Jones [42] method is applied to keep only the *H* most recent observations and using only these observations in the testing procedure. Whenever a new observation arrives, $W_{q}$ and $P_{0,q}$ are computed, and then the longest element is removed from the *H* most recent observations, and the latest value is added. In this way, the breakpoint ϑ calculated by GLR test is limited to the latest *H* data. This method does not ignore all the information outside the window, which not only has statistical significance but also makes the calculation faster. 

Assuming no change occurs, the average number of observations received before a false positive detection is equal to 1/α, where α is the specified probability of an erroneous signal. This quantity is referred to as the average run length (ARL) [43]. The calculation of ARL is a computationally expensive procedure but it only needs to be carried out a single time, and the values can then be stored in a look-up table. We use the Change Point Model (CPM) package [43] in the implementation of GLR control process algorithm, which includes some pre-calculated thresholds for specific ARL, because the control threshold is related to the selection of ARL and takes a lot of computing time.

### 3.4. The Overall Approach of Concept Drifts Detection

The above three modules constitute the proposed method. The origin series data need to be decomposed based on a segment of time series, therefore a sliding window is required. If the window size is too small, it will contain less information, and a larger window will miss catching some local behaviors. Actually, there is not a general way to determine the length of window size, which is related to features of time series. For instance, the window size of data deriving from medical field may be considerably different from the one from financial field. Therefore, the size of the sliding window can be selected according to the prior knowledge in the actual application scenes. 

With the addition of new observations, time series data in the window is decomposed by the extrema symmetric extension EMD method. When drift occurs, it will inevitably lead to changes in the original time series. Since IMFs are the characteristic expressions of the original time series in various time scales, the changes in the internal structures and complexity of IMFs would correspondingly occur. From the above discussion, we can see that, when drifts occur, although the changes are difficult to be directly observed from the original data, the variance and mean of IMF’s entropy have significantly changed. Therefore, in order to detect this change in the environment of streaming data, we introduce a GLR-based statistical process control method. Through GLR statistical test, the breakpoint that maximizes the GLR statistics can be found out. Then, one can judge whether the condition of drifts is reached by comparing GLR statistics with the predefined control threshold. When the drifts are detected, the detector will start again from the next observation value of the detection point. The overall ETFE Algorithm proposed is shown in Algorithm 1. And the implementation code of this algorithm has been uploaded [44].

**Algorithm 1** The overall algorithm of ETFE**Input**: data stream *x*_1_, *x*_2_, … **Initialization**: Initialize the parameters of the specified entropy, the size of sliding window H, the threshold of control limit $\delta_{q}$1**foreach** observation $x_{i}$ in stream do2 **if**
i<H then3  sliding window append $x_{i}$4  **continue**5 **else**6  sliding window append $x_{i}$7 imfs ← EMD({$x_{1},x_{2},\ldots ,x_{w}$}) /* use EMD with the data in sliding window */8 entropy value ← Entropy({imfs}) /* use the specific entropy method to calculate the entropy value of imfs */9 update the interim parameters of GLR with entropy value 10 calculate the GLR test statistic 11 $G_{max,q\leftarrow max_{\vartheta }G_{\vartheta ,q}}$ /* GLR test is used for finding the change point *ϑ* */12 **If**
$G_{max,q}\leq \delta_{q}$ then 13  There is no evidence of drift occurs 14 **else**15  There is evidence of drift occurs16  drift detection position ← ϑ17  drift detection time ←
*i*18  restart from the next observation

From the Algorithm 1, one can see the time complexity mainly lies in the computations of EMD, entropy and GLR test statistic. EMD is widely used in data stream processing because of its low time complexity [28]. The time cost of EMD lies in the generation of IMFs in each iteration, and its time complexity is O(nlogn), where *n* is the length of sliding windows. Here, only the first two IMF are used in the proposed approach. In the calculation of entropy, it is necessary to compare the relations among the reconstructed subsequences, so the time complexity is $O\left(n^{2}\right)$. GLR test statistic is calculated based on the latest window, and the time complexity is O(n). From the above analysis, one can see that time consumption is related to the size of sliding window. Meanwhile, the decomposition and the calculation process of entropy and GLR test statistic are carried out on the data in each sliding window, so the space complexity is also related to the size of the window as O(n), where the sliding window approach is known for avoiding memory cost. Therefore, the proposed algorithm is adequate for real-time streaming data processing. 

Through the analysis of the space and time complexity of the proposed algorithm, it can be seen that the proposed algorithm can be fully applied to the big data scene including high frequency with high volumes, where the detection of concept drifts in the real-time data flow can be achieved. Therefore, the proposed model can be implemented in some applications, such as monitoring abnormal price fluctuation caused by manipulation in financial derivatives market, change of data distribution caused by machine faults in industrial production and the attack of patients, etc. 

## 4. Performance

In this part, a full evaluation of the proposed method is carried out. Firstly, six entropy methods are involved to make a brief comparative study, by which one can intuitively observe the feasibility of scheme. Secondly, by using synthetic data sets, the effectiveness of the proposed method is validated. Thirdly, the real EEG data sets are used to achieve the further verification.

### 4.1. The Evaluation of Various Entropy Methods

**Figure 2:**
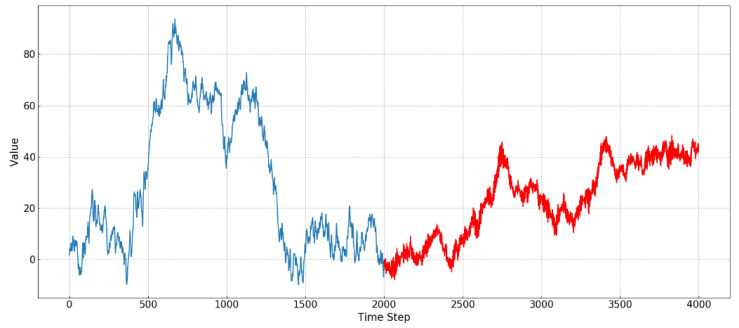
Synthetic time series data consisting of two autoregressive processes.

Two autoregressive processes $x_{t}=1.5x_{t-1}-0.4x_{t-2}-0.3x_{t-3}+0.2x_{t-4}+w_{t}$ and $x_{t}=-0.1x_{t-1}+1.2x_{t-2}+0.4x_{t-3}-0.5x_{t-4}+w_{t}$ are used to create a sequence of data over a period of time, and the synthetic series is shown in Figure 2.

**Figure 3:**
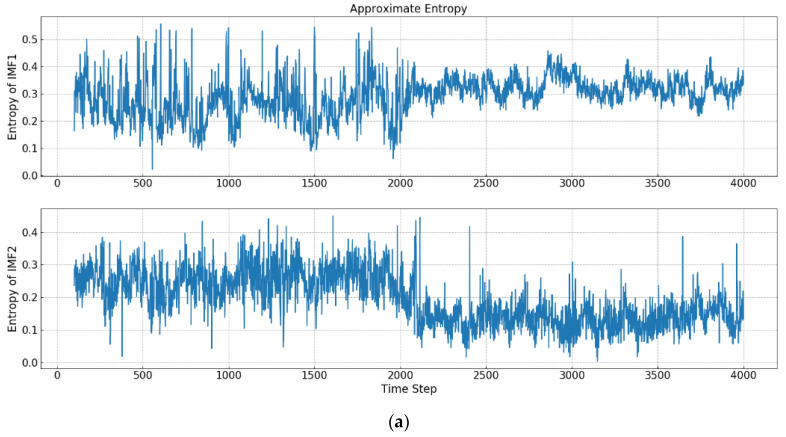

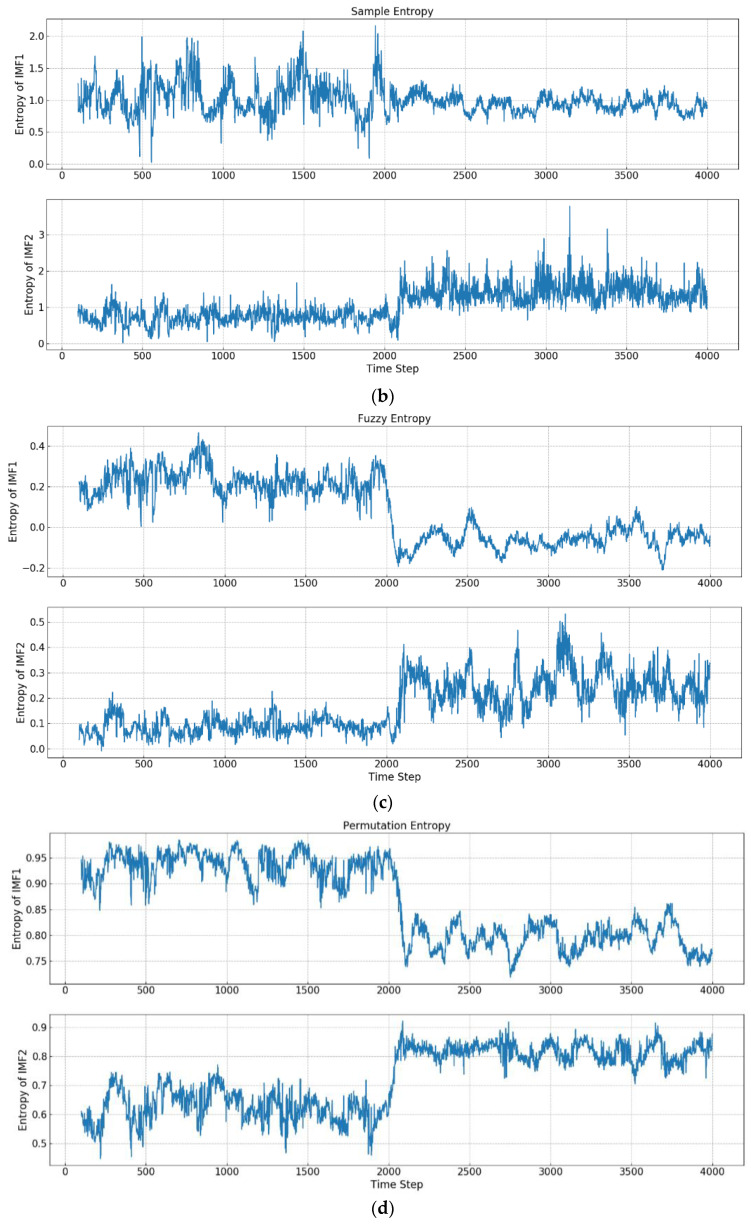

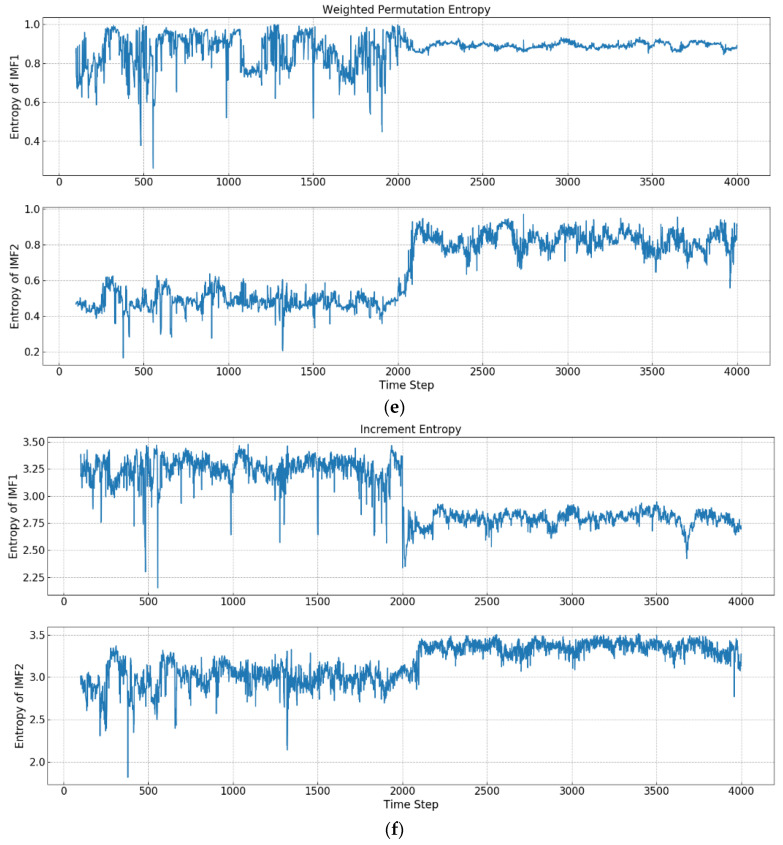
Entropies of intrinsic mode function 1 (IMF1) and intrinsic mode function 2 (IMF2). (**a**) IMF-ApEn; (**b**) IMF-SampEn; (**c**) IMF-FuzzEn; (**d**) IMF-PeEn; (**e**) IMF-WPeEn; (**f**) IMF-IncrEn.

Two autoregressive processes represent two different concepts of time series, and the length of each phase is 2000. As shown in Figure 2, the process of concept drift is simulated by combining two synthetic sequence data, in which distinct concepts are displayed in different colors. As a result of the fluctuations of two time series being similar, it is difficult to be directly detected from the original data. By decomposing the synthesized data, IMFs with different frequency characteristics can be obtained. By using the entropy method, the structure and complexity of each IMF can be quantified. Figure 3 shows the results of IMF1 and IMF2 using different kinds of entropy.

In this group of experiments, IMF1 and IMF2, i.e., the two highest frequency IMFs, are used, where a sliding window with size 100 is set up. Whenever new observation enters, the sliding window moves forward one unit. By transforming the original time series, the entropy change of IMF1 and IMF2 can be seen after 2000 points, where the concept drift occurs and the distribution of data begins to change. 

As to IMF-FuzzEn, it shows that IMF1’s entropy fluctuates around 0.2 in the first concept. After 2000 points, IMF1’s entropy declines significantly and maintains around −0.1. IMF2’s entropy maintains the fluctuation around 0.1 in the first concept. After the first 2000 points, IMF2’s entropy experiences a significant upward change, and maintains around 0.25. It can be seen that the occurrences of the concept drifts will lead to the changes of the structure and complexity of time series in different time-domain features. Since the frequency of IMF1 is higher than the one of IMF2, IMF1 reveals more complex fluctuation patterns and is sensitive to the change of time series. Therefore, when the concept of original time series changes, the entropy of IMF1 can provide a reflection earlier than the one of IMF2. The same situation is also reflected in IMF-PeEn and IMF-IncrEn. 

In IMF-SampEn, after 2000 points, although the mean value of IMF1’s entropy has not obviously changed, the variance reflects large fluctuations, where the variance of IMF1’s entropy becomes smaller and that of IMF2 becomes larger. Similarly, the change of high-frequency IMF1 in ApEn occurs earlier than that of IMF2.

From the results of IMF-WPeEn, one can see that after 2000 points, the mean and variance of the entropies of both IMF1 and IMF2 have changed. The mean of the entropy of IMF1 has increased, but the variance has decreased. Meanwhile, the mean and variance of the entropy of IMF2 have increased. Similarly, the change of IMF1 is earlier than that of IMF2.

From the above results of IMF-Entropy, it can be concluded that, when concept drift occurs, the entropies of IMFs will change in mean, variance, or both. In addition, from the view of entropy, the change of higher frequency IMF is earlier than that of lower frequency IMF, which means that high frequency IMF is more sensitive to the change and low frequency IMF will need a certain time delay to catch the change. Such a mechanism can filter the anomalies or noises in original data. Therefore, the features extracted by the calculation results of IMFs’ entropy can better reflect the concept change of data and have more robustness.

### 4.2. Experiments in Synthetic Data

Although there are many studies on concept drift, the data used for concept drift is mostly based on supervised classification algorithms, and the data set aimed for studies of concept drift in time series is still lack. In order to determine the breakpoints of concept drift and to measure the effectiveness of detection algorithm, synthetic data is also an effective method. Due to the particularity of time series, there is a lack of benchmark data set for concept drift detection of time series in real environment. In this work, the artificial data set in [17] are applied, which contains the time series with concept drifts. In order to simulate the concepts of time series, time series is created using the autoregressive process, that is, time series are represented as $x_{t}=a_{1}x_{t-1}+a_{2}x_{t-2}+,\ldots ,+a_{p}x_{t-p}+w_{t}$, where $w_{t}$ is white noise and subjects to a normal distribution $w_{t}\sim N\left(0,\sigma^{2}\right)$, is the coefficient of the autoregressive model. The standard deviation $\sigma^{2}$ of $w_{t}$ and the autoregressive coefficient $a_{p}$ are shown in Table 1. The data set consists of 120 time series: (1) AR(4) time series, which are affected by AR coefficient and standard deviation of white noise. (2) AR(6) time series, which are affected by AR coefficient and standard deviation of white noise. (3) AR(p) time series, which are affected by order p, AR coefficient, and white noise standard deviation. Each group of data consists of 40 pieces of time series data, each of which has a length of 12,000 points and is composed of 4 concepts. Drifts are achieved by changing the parameters.

According to the common configuration, the parameters of the six entropies are set to be shown in Table 2, where std is the standard deviation of the time series, and the parameter τ in PeEn and WPeEn represents the embedding time delay and the parameter *φ* in IncrEn represents the precision of the fluctuation amplitudes. The sliding window size is 100, the ARL is 200, which is equivalent to the significance level α = 0.95, and the startup is set to be 10% of the total sequence length. It should be noted that we do not pre-process the original data, such as normalization or standardization, so that there is no prior knowledge and can better simulate data flow in the real environment.

**Figure 4:**
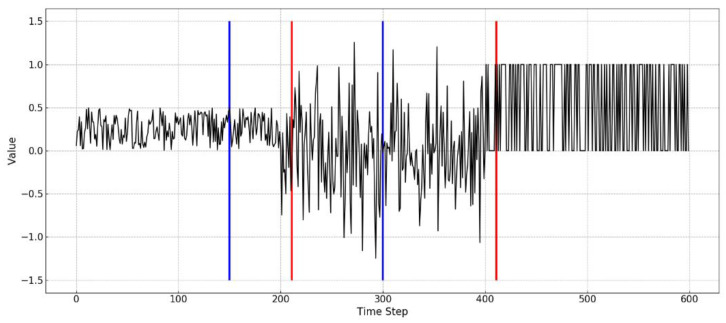
An example of alarms for a data stream (red lines indicate true alarms and blue lines indicate false alarms).

In order to verify the effectiveness of the proposed algorithm in synthetic time series, four metrics, including detection delay, detection position offset, false alarms, and miss detection numbers are implemented, where detection delay represents the number of delay instances between detection time and the occurrence time of drift, detection position offset represents the number of instances between the detection position and the actual drift position, false alarms represents the number of false alarms and miss detection numbers represents the number of true alarms missed by the detector. An example is shown in Figure 4, where the blue line represents the false detection and the red line represents the correct detection. 

In the experiments, the proposed method runs in 120 time series data, each of which runs 30 times. The statistical results obtained by IMF1 and IMF2 are shown in Table 3 and Table 4 in the form of mean ± standard deviation.

In the experiments, the proposed ETFE combining with six kinds of entropy methods are evaluated, the results of which would compare with the existing detection algorithms proposed in [17,23]. The parameter configurations of FEDD, ELM_ECDD, ELM_DDM, and ELM_PHt are the same as those in [17]. The differences of the detection delays between FEDD and ETFE are not obvious, but the proposed algorithm has a fewer detection position offset, which makes a great help for the drift position location in specific production. The proposed ETFE is different from static data detection, and therefore the detection process will be affected by local data, which results in a larger number of false alarms comparing with the five comparisons. However, missing warnings of ELM_DDM, ELM_ECDD, and ELM_PHt are higher than that of the proposed method. In actual application, the harm caused by missing alarms is much serious than that of false detection.

In the actual application, the appropriate entropy method can be selected according to the intrinsic structure of the data to be tested. If the regularity or similarity is present in the time series, the approximate entropy or sample entropy may be selected; fuzzy entropy can be selected when the data are stable or insensitive to parameter selection; when one pays attention to the order relation within the data, the permutation entropy or the increment entropy can be chosen. If one needs to consider fluctuation scale within the data and capture the anomalies, the weighted permutation entropy is the appropriate one.

In addition, from the results of ETFE detection using IMF1 and IMF2, one can obtain that, the detection delay and detection offset of IMF2 are normally higher than those of IMF1, which shows that IMF2, as a low-frequency feature, is less sensitive to time series changes compared with IMF1. And, judging from the number of false alarms, false alarms in IMF2 are less than that those in IMF1, which shows that IMF2 as a low-frequency feature is slightly affected by noise or anomalies. Moreover, the number of miss detection numbers in IMF2 is higher than that in IMF1, which also shows that IMF2 is not sensitive to data changes. Therefore, when IMF2 is used to implement detection, some drifts with slight changes may miss. Even so, the number of missing warnings using IMF2 remains at a very low level. Based on the above results, in the practical application, the high frequency IMFs can be used as a low-delay detection, while the low frequency IMFs can be used as a follow-up drift confirmation, which can make the results more robust and practical.

### 4.3. Experiments in Real Data

The real data applied is a dataset of neonatal EEG recordings and seizure annotations [45]. Neonatal epilepsy is a common emergency in neonatal intensive care unit. The data set contains EEG records from newborns and the labeling of EEG by human experts. EEG records are recorded from 79 newborns in the Neonatal Intensive Care Unit of Helsinki University Hospital. The median duration of these EEG records is 74 minutes (IOR: 64–96 minutes). In the data set, each expert commented on an average of 460 epileptic seizures, including 39 neonatal seizures and 22 non-epileptic seizures by consensus. Detailed data set information can be referred to [45].

In the dataset of neonatal EEG recordings and seizure annotations, not all EEG data are labeled by experts, data from the EEG dataset containing the annotations of the experts are selected. In addition, since the opinions of three experts are not uniform for some periods of onset, in order to ensure the consistency of the expert labeling, 30 periods of data with annotations of three experts are chosen. The applied data sets are shown in Table 5, where Data is the EEG record of selected patients containing epileptic seizures, Annotated Period is the annotation of three experts A, B, and C for epilepsy detection during seizures, and Selected Period is a period of time that contains three experts’ annotation periods. The length of onset time is about 1/3 of the selected time period, which will be used as the data for the effectiveness test of the proposed method.

**Figure 5:**
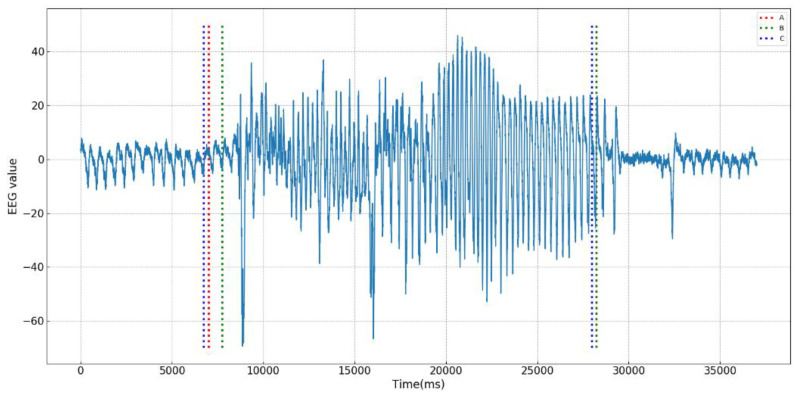
EEG data with expert annotations.

Figure 5 shows a sample of EEG data selected, and the annotations of the experts A, B, and C on the epileptic seizures are indicated by dotted lines in three different colors.

One can observe that the change of EEG data mainly occurs in amplitudes of sequence data. Since the weighted permutation entropy and the increment entropy are more sensitive to the changes of data amplitudes, they are used in the group of experiments. The parameters of WPeEn and IncrEn are the same as those of the previous experiments. The size of sliding window is set to be 100, moving forward 5 units at a time. In the setting of GLR parameters, startup is 20% of the total data length and ARL is 200, which is equivalent to the significance level α = 0.95.

The data stream of EEG data cannot obtain the labels in real time so it is impossible to directly use the supervised detection method. Therefore, in the comparative experiments, the algorithm proposed in [23] is used, where ELM is used to establish a regression model for time series. ELM is a regression model widely used in time series prediction and has strong generalization ability. The regression error $\hat{y}-y_{true}$ is assumed to meet the normal distribution, and the regression errors are monitored by using Drift Detection Method (DDM) [10], Early Drift Detection Method (ECDD) [11] and Page-Hinkley method (PHt) [6]. ELM-DDM, ELM-ECDD, and ELM-PHt have similar application scenarios in concept drift detection, therefore, they are applied for the comparative study.

In order to verify the effect of the proposed method, Cohen’s kappa consistency test [46] is used to calculate the test results obtained by all methods and the annotations of three experts. The Kappa value is calculated in seconds by unifying the unit of expert labeling and the results obtained by all methods, and then the whole EEG records used in the experiment are averaged. All the methods used in the experiments are used to determine the onset interval by monitoring the concept drift in real time. The results presented are kappa values and the corresponding 95% confidence interval obtained by bootstrap. In addition, all methods are compared by false alarm numbers and miss detection rates.

From Table 6, the Kappa values of the detection results of ETFE_WPeEn and ETFE_IncrEn are significantly higher than those of the other three methods. On the one hand, the time-domain features of EEG can be extracted and denoised after decomposition, so as to filter the interference of noise and timely capture the frequency change at the time of onset. On the other hand, the WPeEn and the IncrEn are sensitive to the structural and amplitude changes of the sequence data. Compared with other entropy, the coarsening results obtained by these two entropies can better reflect the changes. And the concept drift can be better detected by the statistical control process based on GLR. Since EEG signals do not change slowly but rapidly during the onset of disease, methods such as DDM and PHt tend to detect abrupt concept drifts more effectively [17], while ECDD is better at the concept drift detection of gradual patterns. Therefore, when detecting EEG data, ELM_DDM and ELM_PHt will more accurately locate the onset of the disease than ELM_ECDD. However, due to the influence of high noise, high frequency, uncertainty, and other factors in EEG data, the overall detection effect of ELM_ECDD, ELM_DDM, and ELM_PHt is not as good as that of ETFE.

**Figure 6:**
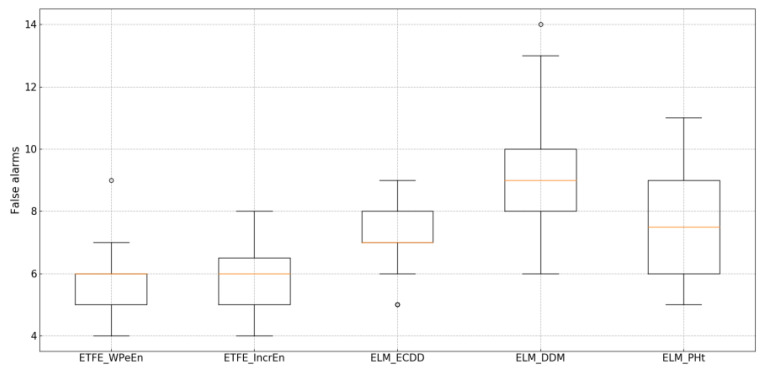
Comparison of false alarms.

Figure 6 shows the effect of these five methods on false alarms. The false alarms of ELM-PHt, ELM-DDM, and ELM-ECDD are significantly higher than those of the proposed algorithm. This is because there are some noises in EEG data, so the fit ability of ELM model is weak when using original data to train ELM directly. Therefore, it is difficult to distinguish the occurrences of concept drifts, which would limit its robustness. The proposed algorithm can obtain features in different time scales, which can play a role in denoising. Furthermore, the features of the original sequence can be transformed by IMF-Entropy. Since the WPeEn and IncrEn are good at capturing the amplitude changes of the sequence. they are used to coarsen the time domain characteristics of the original sequence. Since a statistical process control method that can capture mean and variance changes, GLR will detect such changes and give early warning in time. 

**Figure 7:**
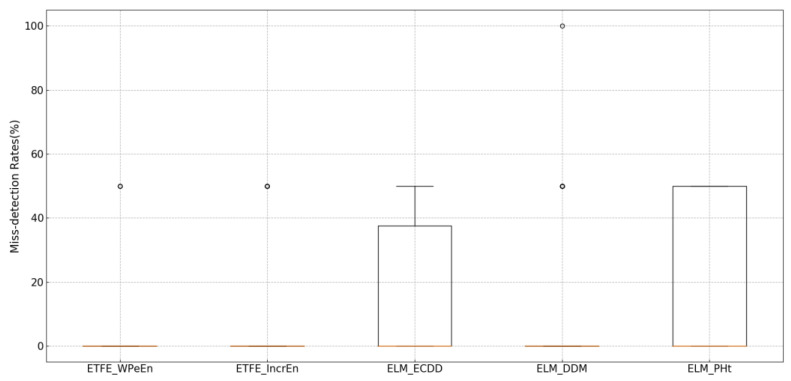
Comparison of miss detection rates.

Figure 7 shows the comparison of five methods in miss detection rates. Since the EEG data to be detected is a segment of data containing epilepsy onset, it is equivalent to three contexts, which means that there are two detection points with concept drifts, where 50% of the detection results mean that only one of the two detection points has been captured. From the display of the results, we can see that the median of all the methods in the box plot of the Missing detection rate is near zero, which indicates that there are few missing detection cases in the detection process. On the one hand, compared with ELM_ECDD and ELM_PHt, the miss detection of ETFE_WPeEn and ETFE_IncrEn only appears as an exception. Meanwhile, the miss detections of ELM_ECDD and ELM_PHt are significantly more than that of the proposed method. On the other hand, compared with ELM_DDM, although it obtains a similar effect, but, ELM_DDM would trigger more error alarms than that of the proposed method.

Generally speaking, experiments show that compared with ELM_ECDD, ELM_DDM, and ELM_PHt, ETFE combined with WPeEn and IncrEn have higher accuracy in determining the onset interval by detecting concept drift, trigger fewer false alarms, and also have lower miss detection rate. 

## 5. Conclusions

In this paper, a novel method called ETFE is proposed for online detection of concept drifts in time series. Firstly, because the real time series data have the characteristics of non-stationary and high noise, the empirical mode decomposition method based on extrema symmetric extension is used to decompose the time series. The time-domain features in different time scales can be effectively extracted and have good signal-to-noise ratio. Secondly, because the concept drift of time series is accompanied by the change of time series structure, the entropy information is used to represent the time-domain characteristics in a coarse-grained way. Finally, when concept drift occurs, the changes of contents in time series will lead to the variation of entropy information. Therefore, the concept drift can be determined by monitoring the changes of the values of mean and variance based on GLR statistical control process.

In the experimental part, synthetic time series data and real data are used to verify the proposed algorithm. As to synthetic time series data, six entropy methods are conducted to discuss the time domain characteristics in different time scales obtained by decomposition. The metrics of detection delay, detection position offset, false alarms, and miss detection numbers are used to verify the effectiveness of the proposed method. In the real data experiment part, the newborn EEG record and epileptic seizure annotation data set are applied, where three existing methods are compared with the proposed method. The results show that our method has better detection results of concept drift with higher robustness. In the further research, when the complexity of time series is analyzed under different time scales, it would be meaningful to introduce multi-scale entropy into this work. In addition, statistical process control methods can be further enhanced to improve the detection of concept drift.

## Figures and Tables

**Table 1:** The parameters of synthetic time series data.

Time Series Group	Concept	$a_{p}$	$\sigma^{2}$
Linear 1	1	{0.9, −0.2, 0.8, −0.5}	0.5
	2	{−0.3, 1.4, 0.4, −0.5}	1.5
	3	{1.5, −0.4, −0.3, 0.2}	2.5
	4	{−0.1, 1.4, 0.4, −0.7}	3.5
Linear 2	1	{1.1, −0.6, 0.8, −0.5, −0.1, 0.3}	0.5
	2	{−0.1, 1.2, 0.4, 0.3, −0.2, −0.6}	1.5
	3	{1.2, −0.4, −0.3, 0.7, −0.6, 0.4}	2.5
	4	{−0.1, 1.1, 0.5, 0.2, −0.2, −0.5}	3.5
Linear 3	1	{0.5, 0.5}	0.5
	2	{1.5, 0.5}	1.5
	3	{0.9, −0.2, 0.8, −0.5}	2.5
	4	{0.9, 0.8, −0.6, 0.2, −0.5, −0.2, 0.4}	3.5

**Table 2:** The parameters of the six kinds of entropy methods.

Entropy Type	Parameters
Approximate Entropy	γ = 3, *r* = 0.2 std
Sample Entropy	γ = 3, *r* = 0.2 std
Fuzzy Entropy	γ = 3, *r* = 0.2 std
Permutation Entropy	γ = 4, τ = 1
Weighted Permutation Entropy	γ = 4, τ = 1
Increment Entropy	γ = 3, *φ* = 2

**Table 3:** Comparisons of ETFE using IMF1 and other detection methods.

Data Set	Method	Detection Delay (Instances)	Detection Position Offset (Instances)	False Alarms	Miss Detection Numbers
Linear 1	ETFE_ApEn	222.31 ± 60.91	45.19 ± 10.61	11.57 ± 2.11	0
	ETFE_SampEn	216.30 ± 71.21	59.05 ± 19.52	10.88 ± 1.95	0.03 ± 0.17
	ETFE_FuzzEn	264.53 ± 89.27	47.33 ± 8.91	12.61 ± 1.67	0.06 ± 0.24
	ETFE_PeEn	249.82 ± 77.36	31.62 ± 9.26	11.38 ± 1.42	0.03 ± 0.17
	ETFE_WPeEn	280.44 ± 81.46	63.51 ± 11.61	10.93 ± 1.38	0
	ETFE_IncrEn	251.71 ± 89.98	34.24 ± 13.56	11.55 ± 2.37	0.03 ± 0.17
	FEDD_cos	197.33 ± 56.67	197.33 ± 56.67	2.47 ± 1.33	0
	FEDD_pear	188.78 ± 43.91	188.78 ± 43.91	2.52 ± 1.17	0.03 ± 0.17
	ELM_ECDD	419.23 ± 97.34	419.23 ± 97.34	3.47 ± 2.37	0.56 ± 0.61
	ELM_DDM	306.66 ± 45.65	306.66 ± 45.65	4.89 ± 1.92	0.43 ± 0.49
	ELM_PHt	487.34 ± 87.40	487.34 ± 87.40	3.56 ± 1.87	0.54 ± 0.51
Linear 2	ETFE_ApEn	300.95 ± 90.40	32.04 ± 12.63	13.61 ± 2.58	0
	ETFE_SampEn	398.20 ± 101.34	56.0 ± 21.42	12.43 ± 1.53	0.03 ± 0.17
	ETFE_FuzzEn	401.20 ± 121.77	67.2 ± 34.76	11.58 ± 1.07	0.03 ± 0.17
	ETFE_PeEn	345.94 ± 77.95	28.0 ± 11.93	10.98 ± 1.53	0
	ETFE_WPeEn	298.35 ± 81.85	44.1 ± 19.80	12.53 ± 1.57	0.03 ± 0.17
	ETFE_IncrEn	387.32 ± 99.29	51.2 ± 21.13	10.12 ± 2.10	0.06 ± 0.24
	FEDD_cos	256.93 ± 87.67	256.93 ± 87.67	2.56 ± 1.11	0.03 ± 0.17
	FEDD_pear	248.34 ± 98.24	248.34 ± 98.24	2.12 ± 1.23	0.03 ± 0.17
	ELM_ECDD	455.86 ± 104.98	455.86 ± 104.98	3.64 ± 1.82	0.54 ± 0.42
	ELM_DDM	411.31 ± 94.56	411.31 ± 94.56	4.78 ± 1.63	0.37 ± 0.38
	ELM_PHt	516.78 ± 132.54	516.78 ± 132.54	3.36 ± 1.66	0.58 ± 0.61
Linear 3	ETFE_ApEn	411.52 ± 121.66	72.9 ± 11.22	10.77 ± 1.41	0
	ETFE_SampEn	512.38 ± 205.30	101.2 ± 29.25	9.89 ± 1.54	0
	ETFE_FuzzEn	503.06 ± 211.45	41.2 ± 19.31	11.55 ± 1.34	0
	ETFE_PeEn	385.57 ± 113.48	52.3 ± 21.54	12.71 ± 1.87	0.03 ± 0.17
	ETFE_WPeEn	431.35 ± 138.35	57.6 ± 14.52	9.78 ± 1.10	0
	ETFE_IncrEn	392.52 ± 177.43	48.3 ± 10.56	11.12 ± 1.16	0
	FEDD_cos	289.78 ± 89.63	289.78 ± 89.63	2.83 ± 1.53	0
	FEDD_pear	304.39 ± 89.10	304.39 ± 89.10	2.32 ± 1.29	0.03 ± 0.17
	ELM_ECDD	501.89 ± 160.35	501.89 ± 160.35	3.66 ± 1.86	0.60 ± 0.22
	ELM_DDM	453.80 ± 174.23	453.80 ± 174.23	4.23 ± 1.43	0.41 ± 0.33
	ELM_PHt	567.32 ± 214.55	567.32 ± 214.55	3.49 ± 1.44	0.53 ± 0.39

**Table 4:** Comparisons of ETFE using IMF2 and other detection methods.

Data Set	Method	Detection Delay (instances)	Detection Position Offset (instances)	False Alarms	Miss Detection Numbers
Linear 1	ETFE_ApEn	382.91 ± 154.34	45.19 ± 11.35	9.13 ± 1.24	0.03 ± 0.17
	ETFE_SampEn	489.02 ± 169.55	69.05 ± 22.44	9.33 ± 1.54	0
	ETFE_FuzzEn	494.17 ± 201.74	78.33 ± 31.52	11.56 ± 2.11	0.06 ± 0.24
	ETFE_PeEn	329.82 ± 139.45	60.13 ± 17.88	11.17 ± 1.33	0.03 ± 0.17
	ETFE_WPeEn	430.21 ± 111.43	71.56 ± 26.43	9.21 ± 1.49	0
	ETFE_IncrEn	442.26 ± 122.43	44.24 ± 10.36	10.08 ± 1.34	0.06 ± 0.24
	FEDD_cos	197.33 ± 56.67	197.33 ± 56.67	2.47 ± 1.33	0
	FEDD_pear	188.78 ± 43.91	188.78 ± 43.91	2.52 ± 1.17	0.03 ± 0.17
	ELM_ECDD	419.23 ± 97.34	419.23 ± 97.34	3.47 ± 2.37	0.56 ± 0.61
	ELM_DDM	306.66 ± 45.65	306.66 ± 45.65	4.89 ± 1.92	0.43 ± 0.49
	ELM_PHt	487.34 ± 87.40	487.34 ± 87.40	3.56 ± 1.87	0.54 ± 0.51
Linear 2	ETFE_ApEn	467.32 ± 144.77	72.30 ± 19.82	9.64 ± 1.34	0
	ETFE_SampEn	472.35 ± 156.81	83.14 ± 21.43	9.89 ± 1.16	0.06 ± 0.24
	ETFE_FuzzEn	367.75 ± 135.65	77.27 ± 13.21	12.57 ± 1.96	0.10 ± 0.3
	ETFE_PeEn	578.85 ± 189.11	48.34 ± 9.87	11.34 ± 1.78	0
	ETFE_WPeEn	414.35 ± 131.32	53.12 ± 12.21	11.56 ± 1.99	0.06 ± 0.24
	ETFE_IncrEn	517.56 ± 176.44	67.33 ± 15.43	12.33 ± 1.87	0.06 ± 0.24
	FEDD_cos	256.93 ± 87.67	256.93 ± 87.67	2.56 ± 1.11	0.03 ± 0.17
	FEDD_pear	248.34 ± 98.24	248.34 ± 98.24	2.12 ± 1.23	0.03 ± 0.17
	ELM_ECDD	455.86 ± 104.98	455.86 ± 104.98	3.64 ± 1.82	0.54 ± 0.42
	ELM_DDM	411.31 ± 94.56	411.31 ± 94.56	4.78 ± 1.63	0.37 ± 0.38
	ELM_PHt	516.78 ± 132.54	516.78 ± 132.54	3.36 ± 1.66	0.58 ± 0.61
Linear 3	ETFE_ApEn	543.87 ± 189.45	89.53 ± 35.43	11.21 ± 1.60	0.03 ± 0.17
	ETFE_SampEn	598.45 ± 197.05	134.23 ± 62.34	9.80 ± 1.42	0.06 ± 0.24
	ETFE_FuzzEn	532.54 ± 156.07	88.23 ± 21.33	11.77 ± 1.50	0
	ETFE_PeEn	433.33 ± 145.67	124.66 ± 65.41	11.46 ± 1.23	0.06 ± 0.24
	ETFE_WPeEn	513.45 ± 173.40	111.76 ± 54.98	9.08 ± 1.09	0.03 ± 0.17
	ETFE_IncrEn	612.24 ± 211.04	156.78 ± 71.37	8.56 ± 1.76	0
	FEDD_cos	289.78 ± 89.63	289.78 ± 89.63	2.83 ± 1.53	0
	FEDD_pear	304.39 ± 89.10	304.39 ± 89.10	2.32 ± 1.29	0.03 ± 0.17
	ELM_ECDD	501.89 ± 160.35	501.89 ± 160.35	3.66 ± 1.86	0.60 ± 0.22
	ELM_DDM	453.80 ± 174.23	453.80 ± 174.23	4.23 ± 1.43	0.41 ± 0.33
	ELM_PHt	567.32 ± 214.55	567.32 ± 214.55	3.49 ± 1.44	0.53 ± 0.39

**Table 5:** EEG data labeled by experts for experiments.

Data.	Annotated Period (s)			Selected Period (s)
	A	B	C	
EEG1	[104, 121]	[96, 122]	[96, 121]	[70, 150]
EEG1	[1179, 1209]	[1178, 1206]	[1179, 1194]	[1150, 1220]
EEG5	[975, 1508]	[993, 1449]	[993, 1446]	[500, 2000]
EEG7	[95, 112]	[97, 106]	[98, 110]	[80, 130]
EEG14	[255, 278]	[254, 282]	[256, 279]	[210, 310]
EEG14	[3331, 3342]	[3330, 3343]	[3330, 3342]	[3310, 3360]
EEG16	[5685, 5707]	[5692, 5707]	[5685, 5706]	[5660, 5730]
EEG17	[2957, 3011]	[2904, 3116]	[2901, 2940]	[2800, 3200]
EEG20	[559, 586]	[563, 584]	[565, 585]	[540, 610]
EEG20	[3827, 3885]	[3824, 3899]	[3827, 3886]	[3760, 3960]
EEG20	[3962, 3980]	[3952, 3985]	[3965, 3980]	[3930, 4010]
EEG25	[3449, 3477]	[3414, 3484]	[3451, 3473]	[3400, 3490]
EEG25	[4792, 4814]	[4767, 4829]	[4792, 4811]	[4750, 4860]
EEG31	[1885, 1964]	[1887, 1966]	[1887, 1966]	[1800, 2040]
EEG31	[2423, 2524]	[2423, 2523]	[2423, 2522]	[2320, 2620]
EEG38	[5367, 5460]	[5369, 5438]	[5369, 5438]	[5300, 5490]
EEG38	[5857, 5886]	[5840, 5889]	[5859, 5885]	[5800, 6020]
EEG44	[294, 375]	[297, 375]	[293, 374]	[210, 450]
EEG44	[644, 661]	[647, 663]	[644, 663]	[620, 690]
EEG44	[2504, 2518]	[2508, 2517]	[2504, 2517]	[2480, 2540]
EEG47	[1841, 1898]	[1841, 1896]	[1832, 1898]	[1790, 1960]
EEG51	[4356, 4684]	[4373, 4679]	[4344, 4663]	[4040, 5000]
EEG62	[1344, 1725]	[1346, 1725]	[1336, 1725]	[940, 2125]
EEG63	[2423, 2526]	[2427, 2528]	[2424, 2519]	[2330, 2630]
EEG67	[751, 780]	[753, 788]	[754, 782]	[720, 810]
EEG67	[1366, 1410]	[1367, 1410]	[1348, 1407]	[1300, 1470]
EEG73	[1429, 1454]	[1429, 1454]	[1429, 1479]	[1380, 1500]
EEG76	[391, 436]	[393, 432]	[386, 435]	[350, 475]
EEG79	[565, 620]	[540, 620]	[566, 620]	[460, 700]
EEG79	[2441, 2494]	[2416, 2490]	[2444, 2493]	[2360, 2570]

**Table 6:** Kappa values and confidence intervals obtained from the test results.

Methods Comparison	Expert A	Expert B	Expert C
ETFE_WPeEn	0.824 (0.705–0.901)	0.802 (0.698–0.895)	0.833 (0.716–0.871)
ETFE_IncrEn	0.815 (0.731–0.897)	0.798 (0.717–0.874)	0.825 (0.729–0.903)
ELM_ECDD	0.655 (0.545–0.713)	0.637 (0.596–0.744)	0.678 (0.530–0.796)
ELM_DDM	0.715 (0.601–0.813)	0.694 (0.612–0.785)	0.723 (0.578–0.849)
ELM_PHt	0.709 (0.604–0.785)	0.661 (0.591–0.762)	0.735 (0.586–0.801)
